# “Kids and Girls”: Parents convey a male default in child-directed speech

**DOI:** 10.1073/pnas.2420810122

**Published:** 2025-03-11

**Authors:** Rachel A. Leshin, Josie Benitez, Serena Fu, Sophia Cordeiro, Marjorie Rhodes

**Affiliations:** ^a^Department of Psychology, Peretsman Scully Hall, Princeton University, Princeton, NJ 08540; ^b^Department of Psychology, New York University, New York, NY 10003

**Keywords:** gender, bias, development

## Abstract

Adults tend to view men (more so than women) as default *people*, with numerous real-world consequences for gender equity. In the United States, the tendency to center men in concepts of *people* develops across middle childhood, yet the specific mechanisms that contribute to it remain unknown. Here, we investigate one subtle but potentially powerful social mechanism: the category labels that parents use to describe boys/men and girls/women in conversations with their children. Across two studies (*N* = 822 parent–child dyads, predominantly from the United States), parents used gender-neutral labels like “kid” or “person” more often to describe boys/men than girls/women and, conversely, used gender-specific labels (e.g., “girl”) more often to describe girls/women than boys/men. These patterns emerged when parents were shown gender-stereotypical girls/women and boys/men (e.g., a girl painting her nails, a boy digging for worms); when parents viewed counterstereotypical stimuli (e.g., a boy painting his nails, a girl digging for worms), the patterns reversed. Our findings illuminate parents’ category label usage as a critical social mechanism that may undergird the development of a male default in a US cultural context, informing efforts to intervene on this process.

When asked to think of a *person*, adults overwhelmingly think of *men*. Adults describe the most typical person they can imagine as male, assume gender-unspecified storybook characters are men, and rate sentences like “He is both a man and a human being” as more redundant than “She is both a woman and a human being” (1–3). This *androcentric* bias is reflected in our collective reality: globally, internet searches for “people” yield more men than women (4, 5), and men remain vastly overrepresented across a host of domains (e.g., politics, media, medicine; 6–8). Who people perceive as the category default has important behavioral consequences: for example, leading participants to perceive men as more typical of a given profession makes them more likely to select male candidates for jobs in that field (4).

How does the expectation that men are default *people* arise? This tendency is not present among young children in the United States, who instead perceive their own gender as most representative. In one study, for example, a racially diverse sample of US children (from middle- to upper-income backgrounds) were asked to choose who to put in a book to teach an alien about people; boys selected pictures of boys/men, whereas girls selected pictures of girls/women. Across middle childhood, however, the strength of girls’ own-gender bias gradually declined, whereas the strength of boys’ remained constant (9). This age-related trend to increasingly view boys as default *people* is evident in the stories children write: in a UK national writing competition, girls favored own-gender protagonists in early childhood but shifted to an equal gender representation by age 12 (while boys continued to write about boys 75% of the time; 10). Such findings converge with the classic “draw a person” task, conducted in Brazil, which finds that by 11 to 12, girls draw boys more often than the reverse (11).

Although androcentrism reflects broad structural, cultural, and historical forces, the *specific* mechanisms that contribute to its emergence in middle childhood—and, in particular, those that may be susceptible to intervention—remain unclear. Here, we seek to uncover one subtle but potentially powerful social mechanism that may undergird (and be leveraged to disrupt) the development of a male default: the category labels that parents use in conversation with their children. From infancy, labels facilitate category learning (12, 13). We thus hypothesized that parents might signal androcentrism via biased labeling in the form of i) increased use of gender-neutral labels^*^ (e.g., “kid,” “person”) for boys vs. girls and ii) increased use of gender-specific labels (e.g., “girl,” “boy”) for girls vs. boys. We tested whether parent language is biased in this manner in a controlled experimental paradigm (Study 1) and in naturalistic open-ended conversations (Study 2).

## Study 1.

**Fig. 1.**
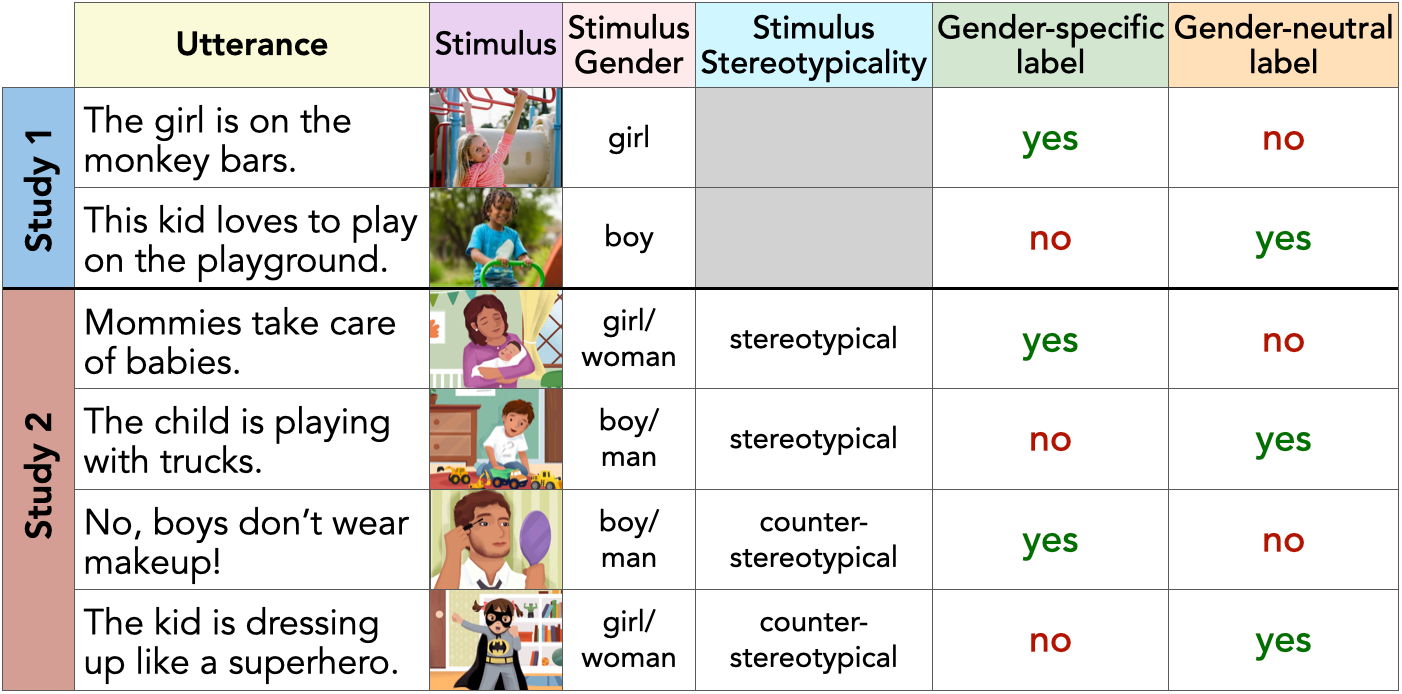
Sample of parents’ language production, stimuli information, and corresponding speech codes.

Parents (*N* = 620) of 4- to 10-y-old children (*M* = 7.24 y; 46% boys, 54% girls; 61% White, 15% Asian, 11% Multiracial, 5% Black/African-American, <1% Other, 8% unreported; 10% Hispanic/Latino overall) were recruited, predominantly from the United States,^†^ to participate in a virtual unmoderated study (see *SI Appendix* for recruitment details). Parents were primarily mothers (of the subset who provided gender information in a follow-up study [*n* = 542], 95% were women) and varied with respect to political ideology (from 1—Very Liberal to 7—Very Conservative, *M* = 3.65, SD = 1.59, range: 1 to 7). In the task, parents were shown four photographs of individual children playing on a playground—a White boy, Black boy, White girl, and Black girl, presented in a randomized order—and asked to provide a caption for each aloud to their children (instructions were provided orally; Fig. 1). Captions were transcribed from participants’ webcam recordings and coded for the presence of gender-neutral and gender-specific labels.

## Results.

**Fig. 2.**
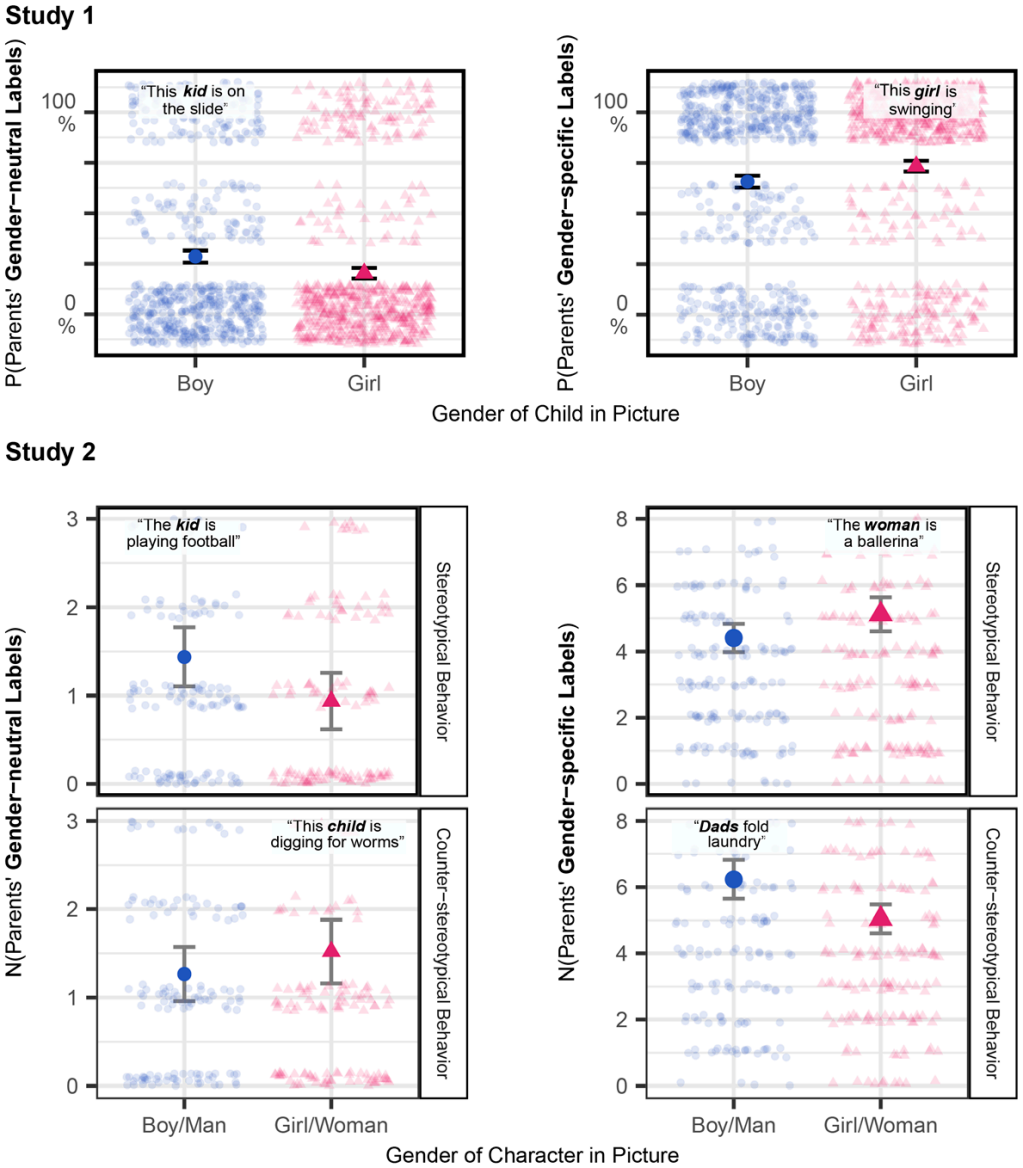
Parents’ category label usage as a function of stimulus gender (*x* axis) and stimulus stereotypicality (facets). Large shapes represent group means, small shapes represent individual response patterns, and error bars are SEs.

Parents were more likely to use gender-neutral labels (e.g., “The kid is sliding”) when describing boys (*M* = 0.28, *95% CI* [0.26, 0.30]) relative to girls (*M* = 0.21, *95% CI* [0.19, 0.22]; main effect of stimulus gender, *b* = −2.27, *SE* = 0.34, *t* = −6.69, *P* < 0.001). Conversely, parents were more likely to use gender-specific labels (e.g., “This girl is swinging”) when describing girls (*M* = 0.73, *95% CI* [0.72, 0.75]) relative to boys (*M* = 0.66, *95% CI* [0.64, 0.68]; main effect of stimulus gender, *b* = 1.77, *SE* = 0.30, *t* = 5.83, *P* < 0.001; Fig. 2). These patterns did not vary by stimulus race (*Ps* > 0.19), child gender (*P*s > 0.12), child age (*P*s > 0.08), or parents’ political ideology (*P*s > 0.27).

## Study 2.

We next tested whether these effects held in more naturalistic parent–child conversations and varied based on the gender stereotypicality of stimuli. As part of an existing longitudinal study conducted on the same remote platform (see *SI Appendix* for recruitment details), 192 parent–child dyads recruited predominantly from the United States^‡^ (95% mothers; 3- to 6-y-old children, *M* = 4.52 y; 55% girls, 45% boys; 70% White, 20% Multiracial, 5% Asian, 2% Black, 2% unreported; 10% Hispanic/Latinx overall) participated in a virtual picture-book-reading task designed to elicit open-ended discussion of gender-related themes. The picture book consisted of 16 pages, each depicting a racially-ambiguous character engaged in a distinct gendered behavior. Pages varied following a 2 (stimulus gender: girl/woman, boy/man) by 2 (stimulus age: child, adult) by 2 (stimulus stereotypicality: stereotypical, counterstereotypical) design and were presented in a random order; each page included a written prompt related to the behavior shown (devoid of labels; e.g., “Who wears makeup?”; Fig. 1) that was visible to families. Conversations were recorded from participants’ webcams and coded for parents’ use of gender-neutral and gender-specific labels. Because category members perceived as more typical elicit category labels more readily (14), we hypothesized that biased labeling reflective of androcentrism would arise more for stereotypical images than for counterstereotypical ones: that is, we predicted that parents’ relative tendency to use gender-neutral labels for boys/men vs. girls/women and gender-specific labels for girls/women vs. boys/men would be strongest for stereotypical trials.

## Results.

Replicating Study 1, for stereotypical behavior, parents used more gender-neutral labels when discussing boys/men (e.g., a boy digging for worms; *M* = 1.44, *95% CI* [1.24, 1.63]) than girls/women (e.g., a girl painting her nails; *M* = 0.94, *95% CI* [0.76, 1.11]; contrast *P* < 0.001), and more gender-specific labels when discussing girls/women (*M* = 5.12, *95% CI* [4.66, 5.58]) than boys/men (*M* = 4.41, *95% CI* [3.98, 4.83]; contrast *P* = 0.001). In contrast, when discussing images depicting counterstereotypical behavior, these patterns reversed: parents generated more gender-neutral labels when discussing girls/women (e.g., a girl digging for worms; *M* = 1.52, *95% CI* [1.30, 1.75]) compared to boys/men (e.g., a boy painting his nails; *M* = 1.27, *95% CI* [1.08, 1.45]; contrast *P* = 0.03; interaction of stimulus gender and stereotypicality, Wald X^2^(1) = 22.34, *P* < 0.001) and more gender-specific labels when discussing boys/men (*M* = 6.23, *95% CI* [5.74, 6.73]) relative to girls/women (*M* = 5.04, *95% CI* [4.66, 5.42]; contrast *P* < 0.001; interaction of stimulus gender and stereotypicality, Wald X^2^(1) = 32.29, *P* < 0.001; Fig. 2). Probing moderation by children’s age and gender, we observed that all patterns described above held when accounting for children’s age (*P*s > 0.64), and bias in parents’ use of gender-neutral labels was consistent across child gender (*P* = 0.18). For bias in parents’ use of gender-specific labels, we observed additional moderation by child gender (interaction of child gender, stimulus stereotypicality, and stimulus gender *P* = 0.049): for pages depicting counterstereotypical behavior, parents of boys did not differ in their use of gender-specific labels for boys/men relative to girls/women (although patterns were directionally consistent with the overall finding), but in all other instances, patterns of bias mirrored those observed in the overall sample and reached statistical significance.

## Discussion

We observed systematic patterns of bias in US parents’ descriptions of boys and girls: parents were more likely to use gender-neutral labels to describe boys vs. girls and gender-specific labels to describe girls vs. boys. This robust bias in parents’ language may potentially serve as a subtle—and modifiable—cue to androcentric thinking in childhood within this cultural context. Parents showed this pattern of biased labeling when characters were depicted engaging in neutral behaviors and stereotypical ones, but these patterns reversed when characters were presented counterstereotypically. Such a reversal may reflect the relative difficulty with which people produce category labels for atypical members (i.e., reflecting the possibility that counterstereotypical behaviors led parents to view boys/men as less typical of *people* and girls/women as less typical of their gender) or the tendency for feminine behavior to be marked by gender more overall (i.e., reflecting the possibility that depictions of feminine behavior—counterstereotypical for boys and stereotypical for girls—led parents to generate more gender-specific language). These and other possibilities will need to be disentangled in future research. Altogether, our findings suggest that bias in US parents’ category label usage may signal the default person as *male*, while also implying *which* boys/men to think of in this manner (i.e., those displaying stereotypical behaviors). Future research should directly assess the link between bias in parents’ labeling (as well as use of related linguistic cues, e.g., generic “he”; 15) and children’s beliefs that boys/men represent default *people*. Our findings provide a critical first step in identifying the roots of androcentric thinking in the United States and, ultimately, suggest how it might be mitigated.

## Materials and Methods

Methods and analyses for Study 1 were drawn from a larger study of parents’ language and beliefs (https://osf.io/wbqjr and https://osf.io/3nrcv); for Study 1, we focused specifically on measures of parent language. Methods and analyses for Study 2 were also drawn from a larger study (https://osf.io/ep82f). Prior to beginning each study, parents consented (and children assented) to providing survey data and webcam video. The Institutional Review Board of New York University (IRB-FY2016-760) approved all consent procedures. Data, analysis scripts, and materials can be accessed at https://osf.io/xjckv/. For more details, see *SI Appendix*.

## Supplementary Material

Appendix 01 (PDF)

## Data Availability

Anonymized quantitative data have been deposited in Open Science Framework (https://osf.io/xjckv/) (16).

## Footnotes

^*^Gender-neutral labels that contained other identity-related content (e.g., occupation labels) were not considered.

^†^Twelve children participated from outside the United States.

^‡^Five children participated from outside the United States.
