# Supplementary material for: “Kids and Girls”: Parents convey a male default in child-directed speech
Source: Proc Natl Acad Sci U S A. 2025 Mar 11;122(11):e2420810122. doi: 10.1073/pnas.2420810122 (PMC11929473; doi:10.1073/pnas.2420810122)
Supplement: Supplementary file 1 — Appendix 01 (PDF) [file pnas.2420810122.sapp.pdf]

PNAS

Supporting Information for

"Kids and Girls": Parents convey a male default in child-directed speech

Rachel A. Leshin[1], Josie Benitez[2], Serena Fu[2], Sophia Cordeiro[2], & Marjorie Rhodes[2]

Corresponding Author: Rachel A. Leshin

Email: rachel.leshin@princeton.edu

This PDF file includes:

Supporting text
SI References

Supporting Information Text

Extended Methods: Study 1

Participants. Data were collected as part of a larger study on parents' language and beliefs (pre-registered at https://osf.io/wbqjr/?view_only=94da6529fb094704bd00f1f8402d051e and [blinded for review; this second pre-registration contains identifying information]) administered on an online platform designed to conduct remote unmoderated research with children and their parents (http://discoveriesinaction.org/; Rhodes et al., 2020). Families were recruited to the platform via in-person recruitment events (e.g., at schools, museums, public parks) and online advertisements (e.g., parenting podcasts, blogs, Facebook ads); all eligible families on the platform with 4- to 10-year-old children were invited to participate. The demographics of participants in Study 1 largely mirror those of the online participant database overall. We administered two separate rounds of data collection for Study 1: the first (*n*=376) was launched and completed in February 2023, and the second (*n*=244) was launched and completed in July 2023. The parent language measures reported in the main manuscript for Study 1 were identical across both phases of study administration. Families consented to providing Qualtrics survey and webcam video data and were compensated with a $10 Amazon Gift Card for completion of the study.

Caption Transcription and Speech Coding. Study sessions were recorded via participants' webcams and uploaded to the online platform at the end of the session. The captioning portion of each experimental session was then transcribed from the video recording by a trained research assistant. In addition to transcribing the captions that parents supplied for each of the four pictures, research assistants also noted instances in which children had interfered with their parents' responses (e.g., interrupting parents, providing a response on parents' behalf). The transcribed oral data file was then compared with the data file containing parents' written responses, which they were asked to log after saying each caption aloud to their child in the study. We used the written data only in instances in which we identified issues with the transcribed data (e.g., parents didn't supply a caption out loud, the audio quality of the video made it difficult to parse the oral caption); in all other instances, we used the oral transcription data. We eliminated any trials on which researcher assistants had identified (via oral transcriptions) evidence that children interfered with their parents' answers (<1% of coded trials).

All parent captions were coded by a trained research assistant for their category label content, and 50% of these were independently coded by a second trained researcher for reliability. Captions were coded for (1) the presence of gender-specific labels (e.g., "boy," "girl," "lady," "sister") and (2) the presence of gender-neutral labels (e.g., "kid," "child," "person," "bud"). All captions containing a gender-specific label received a "1" for this construct (otherwise, they received a "0"), and all responses containing a gender-neutral label received a "1" for this construct (otherwise, they received a "0"). Coding for each caption was not mutually exclusive; thus, it was possible for a given caption to receive a "1" for both constructs (e.g., "This girl is swinging. Looks like the kid's having a fun time"), and it was also possible for a given caption to receive a "0" for both constructs (e.g., "She's on the swing"; "Having fun at the playground"; see https://osf.io/xjckv/?view_only=c06e89c0a81b4ccabba52c0e13929f5c for full coding manual). Coders' reliability was excellent (percent agreement: 100%).

Measure of Parents' Political Ideology. To assess parents' political ideology, we used a brief self-report measure. Parents were asked "How would you rate your overall political views?" and responded on a sliding scale that ranged from 1 (labeled as "Very liberal") to 7 (labeled as "Very conservative").

Primary Analyses: Analytic Strategy. All analyses were conducted in *R* (version 4.4.0). To test our two primary questions (i.e., Does parents' use of gender-neutral labels vary by stimulus gender? Does parents' use of gender-specific labels vary by stimulus gender?), we ran two generalized linear models using the *lme4* package (Bates et al., 2015) and specified a binomial distribution. As pre-registered, in each model we regressed parents' score–that is, a binary value reflecting whether parents' response for a given trial contained (coded as 1) or did not contain

(coded as 0) the type of category label in question–onto the gender of the stimulus, the race of the stimulus, and their interaction, with subject ID entered as a random effect. We additionally controlled for the round of data collection (February 2023 or July 2023) and the order in which parents saw each stimulus (1-4) by entering each of these variables as covariates to our models (neither of these variables significantly interacted with stimulus gender). To test for moderation by child gender, child age, and parents' political ideology, we added each of these variables, one at a time, as a fixed predictor allowed to interact with stimulus gender (we eliminated stimulus race from these analyses given that we found no main or interactive effects in our primary models) and again controlled for the round of data collection and stimulus order. We report *b*-values, *t*-values, standard errors, and *p*-values from our models in the main text; means reflect parents' average likelihood of using each type of label for boy and girl stimuli, with 95% confidence intervals.

Additional Analyses. In the main manuscript, we describe the findings from Study 1 in the context of our primary research question by assessing parents' use of gender-neutral labels for girls vs. boys (main effect of stimulus gender *b*=-2.27, *SE*=.34, *t*=-6.69, *p*<.001) and their use of gender-specific labels for girls vs. boys (main effect of stimulus gender, *b*=1.77, *SE*=.30, *t*=5.83, *p*<.001). In the sections below, we provide details related to parents' use of gender-neutral vs. gender-specific labels, as well as parents' use of gender-neutral vs. gender-specific labels for boy stimuli and girl stimuli separately (subsection I). We also report a separate set of analyses in which we assess, in a single linear mixed-effects model, parents' relative likelihood of using gender-specific vs. gender-neutral labels on each trial (expressed as a difference score) as a function of the variables tested in our primary analyses (we note that, unlike in our main analyses, we do not specify a binomial distribution here given that difference scores are not dichotomous; subsection II). Although parents' use of each type of label is not entirely interdependent, these analyses help to provide additional nuance to our main results.

I. In general, parents tended to use gender-specific labels (*M*=.70, *95% CI* [.67, .72]) more often than gender-neutral labels (*M*=.24; *95% CI* [.22, .27]; main effect of label, *b*=-1.96, *SE*=.07, *t*=-30.00, *p*<.001). These patterns were corroborated for both pictures of girls and pictures of boys. For pictures of girls, parents used gender-specific labels 73% of the time (*95% CI* [.70, .77]) and gender-neutral labels 20% of the time (*95% CI* [.17, .23]; main effect of label, *b*=-2.38, *SE*=.10, *t*=-24.51, *p*<.001). For pictures of boys, parents used gender-specific labels 66% of the time (*95% CI* [.62, .69]) and gender-neutral labels 29% of the time (*95% CI* [.25, .32]; main effect of label, *b*=-1.57, *SE*=.09, *t*=-17.68, *p*<.001).

II. Parents' relative tendency to describe children with gender-specific vs. gender-neutral labels was stronger for images of girls (*M*=.52, *95% CI* [.50, .55]) than images of boys (*M*=.38, *95% CI* [.34, .41]; main effect of stimulus gender, *b*=.16, *SE*=.03, *t*= 6.17, *p*<.001). As with the effects reported in the main manuscript, this pattern did not vary as a function of stimulus race (*p*=.54), child gender (*p*=.13), children's age (*p*=.64), or parents' political ideology (*p*=.77). Thus, the findings from our main analyses that *separately* probe parents' use of gender-specific and gender-neutral labels as a function of stimulus gender are corroborated when we assess parents' *relative* use of each type of label.

Extended Methods: Study 2

Participants. Data were obtained from an existing longitudinal study on parent-child natural language about gender that took place on the online platform described above (pre-registered at https://osf.io/ep82f/?view_only=f036f7f88cd448178c4be4d1fcf2c3cc). As in Study 1, families were recruited to the platform via in-person recruitment events in New York City (e.g., schools, museums, public parks) and online advertisements (e.g., parenting podcasts, blogs, Facebook ads). The demographics of participants in Study 2 largely mirror those of the online participant database overall. At the time of the initial study launch in April 2020, all eligible families registered on the platform who had 3- to 5-year-old children were invited to participate from their home computers. Families consented to providing Qualtrics survey and webcam video data and were compensated with a $10 Amazon Gift Card for successful completion of the study.

Natural Language Transcription and Speech Coding. Parent-child conversations during the picture book task in Study 2 were recorded via participants' webcams and uploaded to the online

platform at the end of the session. Participant sessions with full study video, audio, and Qualtrics data were transcribed and speech-coded at the utterance-level in a series of iterative passes by a team of research assistants using the Datavyu Video Coding platform (2014; see https://osf.io/xjckv/?view_only=c06e89c0a81b4ccabba52c0e13929f5c for a full coding manual). For the purposes of Study 2, an utterance was defined as any unit of speech distinguished by grammatical closure, pause, or intonation (e.g., the statement “Look at that boy. He’s playing football.” would be coded as two separate utterances). All coders were trained by the second author using sample videos; all valid session videos (n = 192, 100.00%) were subjected to review by a second coder who addressed any discrepancies in transcription and speech coding by discussing with the coding team.

To account for natural deviations in families’ conversations during the picture book task, all Study 2 videos were first subjected to focus coding. Utterances that reasonably related to the task were coded as “on-task” (focused), while utterances that sought clarification (e.g., “Huh?”, “Yeah?”) or did not relate to the picture book (e.g., “Stop touching that”, “Next page”) were coded as “off-task” (not focused); overall, 88.28% of all utterances were coded as “on-task” (focused). Next, on-task utterances were coded to determine whether they explicitly referred to a person/people (e.g., “Yes, that girl has a nice dress”, “That’s what they play at my school”, including references to third-parties such as “Batman”) or not (e.g., “What color is that car?”); overall, 59.87% of on-task utterances were coded as person-referencing. All on-task person-referencing utterances were then coded for parents’ use of (1) gender-neutral and (2) gender-specific labels.

Primary Analyses: Analytic Strategy. Given the open-ended nature of the picture book reading task, it was possible that parents could produce any number of utterances on a given page. As such, analyses of parents’ natural language were conducted using a count-based approach that considered participants’ decisions to use gender-neutral and gender-specific labels in a certain context. In particular, our pre-registered primary analyses examined whether the number of times parents used gender-neutral and gender-specific labels varied as a joint function of the gender (girl/woman, boy/man) and stereotypicality (stereotypical, counter-stereotypical) of the stimulus depicted on each page. Data were examined in *R* (version 4.1.2) using generalized linear regression models in the *glmmTMB* package (Brooks et al., 2023), specified for a poisson distribution. All mixed-effects models included subject ID as a random effect. We report and interpret Wald X^2 tests from the “Anova” function as indicators of significant effects; means are presented as the average number of utterances per stimulus-type in the picture book, with 95% confidence intervals. All significant interactions involving stimulus gender and stereotypicality were analyzed using pairwise contrast t-tests from the *emmeans* package (Searle et al., 1980). Lastly, to test for moderation by child gender and child (mean-centered) age, we added each of these variables into separate exploratory models as an additional fixed predictor, allowed to interact with all other variables in the initial model (stimulus gender and stereotypicality).

Additional Analyses. In the main manuscript, we describe the findings from Study 2 in the context of our primary research question by assessing parents’ use of gender-neutral labels (interaction of stimulus gender and stereotypicality, Wald $X^2(1)=22.34$, $p<.001$) and gender-specific labels (interaction of stimulus gender and stereotypicality, Wald $X^2(1)=32.29$, $p<.001$) when discussing pictures of girls/women *vs.* boys/men, both when depicted stereotypically and counter-stereotypically. In the sections below, we instead examine parents’ gender-neutral and gender-specific label usage on pages depicting gender stereotypical *vs.* counter-stereotypical behavior, both for pictures of girls/women and boys/men (subsection I). We additionally describe parents’ overall use of gender-specific and gender-neutral labels, collapsing across both stimulus gender and stereotypicality (subsection II). Finally, we report a separate set of analyses in which we test, in a single linear mixed-effects model, parents’ *relative usage* of gender-specific vs. gender-neutral category labels within each utterance (expressed as a difference score) as a function of the variables tested in our primary analyses (sub-section III; note that, unlike in our main analyses, we do not specify a poisson distribution given that differences scores do not constitute count-based data). We highlight, as above, that parents’ use of each type of label is not

interdependent—this is particularly true in Study 2, given the open-ended nature of the design—but nonetheless include these difference score analyses for added nuance.

I. For stimuli showing girls/women, parents produced more gender-neutral labels when discussing gender counter-stereotypical behavior (e.g., a girl digging for worms; *M*=1.52, *95% CI* [1.30, 1.75]) compared to stereotypical behavior (e.g., a girl painting her nails; *M*=0.94, *95% CI* [0.76, 1.11]; contrast *p*<.001); however, parents' use of gender-specific labels for girls/women did not differ by stereotypicality (stereotypical behavior, *M*=5.12, *95% CI* [4.66, 5.58]); counter-stereotypical behavior, *M*=5.04, *95% CI* [4.66, 5.42]; contrast *p*=.73). In contrast, for stimuli showing boys/men, parents' use of gender-neutral labels did not vary by stereotypicality (stereotypical behavior, *M*=1.44, *95% CI* [1.24, 1.63]; counter-stereotypical behavior, *M*=1.27, *95% CI* [1.08, 1.45]; contrast *p*=.15), but their use of gender-specific labels did: parents used more gender-specific labels for boys/men when discussing counter-stereotypical behavior (*M*=6.23, *95% CI* [5.74, 6.73]) relative to stereotypical behavior (*M*=4.41, *95% CI* [3.98, 4.83]; contrast *p*<.001). Thus, in summary, parents' use of gender-neutral (but not gender-specific) labels differed when describing stereotypical vs. counter-stereotypical girls/women, while their use of gender-specific (but not gender-neutral) labels differed when describing stereotypical vs. counter-stereotypical boys/men.

II. With respect to parents' overall use of category labels–collapsing across stimulus gender (girl/women, boy/men) and stereotypicality (stereotypical, counter-stereotypical)–parents produced more gender-specific labels (*M*=20.80, *95% CI* [17.98, 23.62]) than gender-neutral labels (*M*=5.16, *95% CI* [3.81, 6.51]; main effect of label, Wald $X^2(1)$=1542.5, *p*<.001) on average.

III. When discussing images depicting stereotypical behavior, parents' relative use of gender-specific vs. gender-neutral labels was greater for images of girls/women (*M*=4.18, *95% CI* [3.70, 4.67]) than images of boys/men (*M*=2.97, *95% CI* [2.50, 3.44]; contrast *p*<.001). In contrast, when discussing images depicting counter-stereotypical behavior, parents' relative use of gender-specific vs. gender-neutral labels was greater for images of boys/men (*M*=4.97, *95% CI* [4.45, 5.49]) than images of girls/women (*M*=3.52, *95% CI* [3.06, 3.98]; contrast *p*<.001; interaction of stimulus gender and stereotypicality, *b*=2.66, *SE*=.49, *t*= 5.43, *p*<.001). This pattern did not vary as a function of child gender (*p*=.42) or age (*p*=.65). Thus, as in Study 1, analyses of parents' *relative* use of gender-specific vs. gender-neutral labels corroborate the effects reported in the main text.

SI References

1. Bates, D., Mächler, M., Bolker, B., & Walker, S. (2015). Fitting Linear Mixed-Effects Models Using lme4. *Journal of Statistical Software*, *67*(1), 1–48. https://doi.org/10.18637/jss.v067.i01
2. Brooks, M., Bolker, B., Kristensen, K., Maechler, M., Magnusson, A., & McGillycuddy, M. (2023). Package 'glmmtmb'. R Package Version, 1(1), 7. https://cran.r-project.org/web/packages/glmmTMB/glmmTMB.pdf
3. Datavyu Team (2014). Datavyu: A Video Coding Tool. Databrary Project, New York University. URL http://datavyu.org.
4. Rhodes, M., Rizzo, M. T., Foster-Hanson, E., Moty, K., Leshin, R. A., Wang, M., Benitez, J., & Ocampo, J. D. (2020). Advancing developmental science via unmoderated remote research with children. Journal of Cognition and Development, 21(4), 477-493. https://doi.org/10.1080/15248372.2020.1797751
5. Searle, S. R., Speed, F. M., & Milliken, G. A. (1980). Population marginal means in the linear model: an alternative to least squares means. The American Statistician, 34(4), 216-221. https://doi.org/10.1080/00031305.1980.10483031
